# Maternal Exercise Prior to and during Gestation Induces Sex-Specific Alterations in the Mouse Placenta

**DOI:** 10.3390/ijms242216441

**Published:** 2023-11-17

**Authors:** Meghan L. Ruebel, Sarah J. Borengasser, Ying Zhong, Ping Kang, Jennifer Faske, Kartik Shankar

**Affiliations:** 1 Microbiome and Metabolism Research Unit, USDA-ARS, Southeast Area, Little Rock, AR 72202, USA; meghan.ruebel@usda.gov; 2 Arkansas Children’s Nutrition Center, University of Arkansas for Medical Sciences, Little Rock, AR 72202, USA; zhongying@uams.edu (Y.Z.); jennifer.faske@fda.hhs.gov (J.F.); 3 Tobacco Settlement Endowment Trust Health Promotion Research Center, Stephenson Cancer Center, University of Oklahoma Health Sciences Center, Oklahoma City, OK 73104, USA; sarah-borengasser@ouhsc.edu; 4 Harold Hamm Diabetes Center, University of Oklahoma Health Sciences Center, Oklahoma City, OK 73104, USA; 5 Department of Pediatrics—Endocrinology & Diabetes, University of Oklahoma Health Sciences Center, Oklahoma City, OK 73104, USA; 6 Division of Genetic and Molecular Toxicology, National Center for Toxicological Research, U.S. Food and Drug Administration, Jefferson, AR 72079, USA; 7 Department of Pediatrics, Section of Nutrition, University of Colorado Denver, Anschutz Medical Campus, Aurora, CO 80045, USA

**Keywords:** maternal exercise, fetal programming, placenta, sexual dimorphism

## Abstract

While exercise (EX) during pregnancy is beneficial for both mother and child, little is known about the mechanisms by which maternal exercise mediates changes in utero. Six-week-old female C57BL/6 mice were divided into two groups: with (exercise, EX; N = 7) or without (sedentary, SED; N = 8) access to voluntary running wheels. EX was provided via 24 h access to wheels for 10 weeks prior to conception until late pregnancy (18.5 days post coitum). Sex-stratified placentas and fetal livers were collected. Microarray analysis of SED and EX placentas revealed that EX affected gene transcript expression of 283 and 661 transcripts in male and female placentas, respectively (±1.4-fold, *p* < 0.05). Gene Set Enrichment and Ingenuity Pathway Analyses of male placentas showed that EX led to inhibition of signaling pathways, biological functions, and down-regulation of transcripts related to lipid and steroid metabolism, while EX in female placentas led to activation of pathways, biological functions, and gene expression related to muscle growth, brain, vascular development, and growth factors. Overall, our results suggest that the effects of maternal EX on the placenta and presumably on the offspring are sexually dimorphic.

## 1. Introduction

The Developmental Origins of Health and Disease (DOHaD) hypothesis posits that the first 1000 days of life (conception through 2 years of age) represents a critical developmental window during which nutritional and environmental influences can potentially impact disease risk throughout life [1,2,3]. Greater pre-pregnancy weight and inter-pregnancy weight gain have been shown to detrimentally impact offspring health compared to siblings born prior to increased maternal body weight [4]. Conversely, children born to mothers with obesity who underwent bariatric surgery were metabolically healthier than their siblings who were born prior to surgery [5,6]. Emerging evidence also suggests that the critical period influencing long-term development likely extends to the preconception period [7,8,9], suggesting both preconception and gestation may be key windows to intervene and reduce detrimental programming in the offspring. Lifestyle modifications commencing prior to conception, such as diet and exercise, are promising interventions to mitigate the intergenerational cycle of obesity.

Clinical studies have reported that maternal exercise during gestation improves pregnancy and birth outcomes [10,11,12] and reduces the risk of delivering an infant with macrosomia as compared to mothers who did not exercise [13,14]. Maternal exercise can also reduce the adiposity of offspring and improve cardiac health and neurobehavioral outcomes in infants [15,16,17,18]. Rodent models of maternal exercise during gestation have demonstrated reduced body weight and adiposity, improved insulin sensitivity, and changes to skeletal muscle, pancreas, liver, brain and adipose tissues in offspring [15,19,20,21,22,23,24,25], with adiposity and insulin sensitivity changes occurring in a sexually dimorphic manner [19,20,26,27,28,29].

Maternal health and environmental insults have been shown to contribute to the persistent programming of offspring metabolic phenotypes. The placenta is thought to be a central mediator of developmental programming, as it is the sole site of nutrient exchanges which is necessary in orchestrating growth and development in utero, as well as a conduit to relay metabolic information about the maternal habitus to the developing offspring [30,31,32]. In the context of maternal exercise during pregnancy, studies have shown changes in morphology, vascularization, nutrient transport, and gene expression of the placenta [15,27,33,34]. It is important to note that most of these studies examined the effects of exercise in addition to a high-fat diet to see how maternal exercise improved high-fat diet/obesity effects. However, a recent study showed that maternal exercise can lead to an increase in placental *Sod3* and vitamin D receptor (*Vdr*) expression independent of maternal high-fat diet and may be linked to metabolic changes in the offspring’s liver [35]. However, these studies did not perform sex-stratified placenta analyses, which may be important as the placenta can demonstrate sexually dimorphic effects with maternal exposures, such as diet. Therefore, there is a critical need for a detailed analysis of alterations in the placenta following maternal exercise in pregnancy.

The present study examined the role of maternal voluntary wheel running commencing 10 weeks prior to gestation, and throughout pregnancy, on placental and fetal liver mRNA expression in late gestation. The purpose of this study was to assess early responsiveness to maternal exercise and examine sex differences in the placenta and offspring. The placenta serves as the principal interface between maternal and fetal circulation and serves as the hub for nutrient sensing and transport, gas exchange, and excretion of waste [36,37]. Thus, by studying the placenta and fetal liver, we sought to better understand the benefits of maternal exercise prior to and during gestation on early molecular programming that may contribute to later metabolic improvements in the offspring. Our findings demonstrated maternal exercise-induced and sex-specific transcriptomic differences that were enriched in pathways that included lipid transport (males), muscle differentiation (females), and development (males and females).

## 2. Results

### 2.1. Maternal Wheel Running Measurements and Phenotypic Characteristics

**Figure 1:**
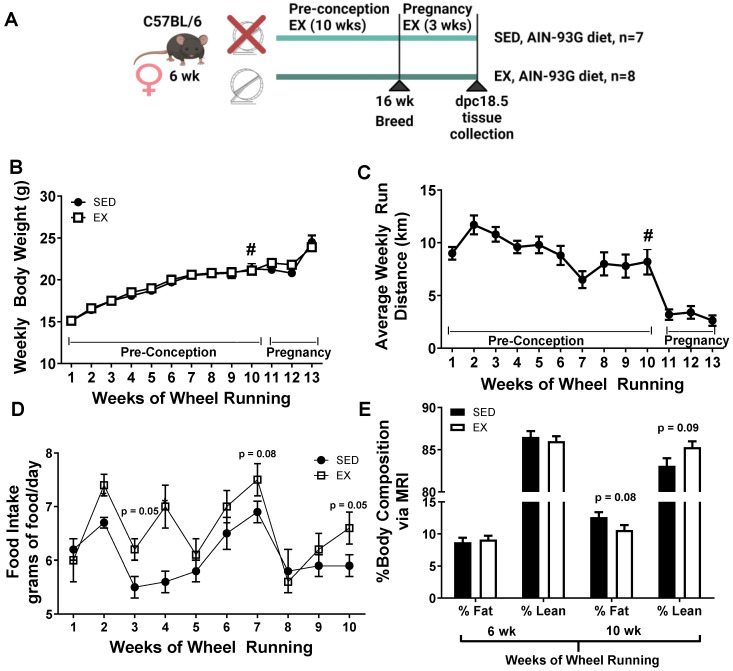
Dam characteristics prior to and during pregnancy from sedentary (SED) (N = 8) and exercise (EX) dams (N = 7). (**A**) Timeline of experimental protocol created by Biorender.com (**B**) Average weekly body weights for SED and EX dams, (**C**) daily run distance for EX dams, (**D**) food intake, and **(E**) percent body fat and lean mass at 6 and 10 wk of wheel running by magnetic resonance imaging (MRI). Data are expressed as means ± SEM. Statistical differences were determined using Student’s *t*-test. # denotes that females were bred with control males starting at week 10 for 4 days.

Maternal voluntary wheel running prior to and during gestation had no effect on body weight, but increased food intake in female dams. Body weights were not different between SED and EX dams as shown in Figure 1B despite EX dams running 7.6 km per day over the 10 weeks of access to running wheels prior to conception (Figure 1C). However, food intake was increased at week 2 and week 4 and a nonsignificant trend was observed towards increases at week 3 (*p* = 0.05), week 7 (*p* = 0.08), and week 10 (*p* = 0.05) in EX dams as compared SED dams, shown in Figure 1D. Body composition was assessed at 6 weeks and 10 weeks of voluntary wheel running. EX dams demonstrated a nonsignificant tendency towards reduced body fat percentage (*p* = 0.07) and a higher percentage of lean mass (*p* = 0.09) after 10 weeks of wheel running as compared to SED dam counterparts (Figure 1E). The data suggest that EX dams increased food intake to account for the increased caloric demands of wheel running to maintain body weight as compared to SED counterparts, allowing for the effects of maternal exercise to be examined independent of maternal weight loss. Moreover, despite increased food intake, voluntary wheel running prevented EX dams from gaining excess adiposity. There were no phenotypic differences between EX and SED dams at the time of sacrifice, dpc 18.5, as shown in Table 1. The only exception was a ~20% increase in circulating triglycerides in EX dams (61.1 ± 3.3 mg/dL vs. 50.7 ± 2.2 mg/dL).

### 2.2. Placenta Characteristics and Fetal Weights

**Figure 2:**
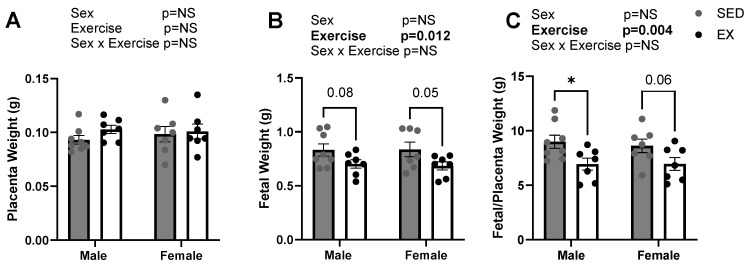
Placental and fetal weights at dpc 18.5 from fetuses from sedentary (SED; N = 8) and exercise (EX) dams (N = 7) divided out by sex. (**A**) Average placental weight, (**B**) average fetal weight, and (**C**) placental efficiency for male and female fetuses born to SED and EX dams. At dpc 18.5 sex-stratified placenta and fetus weight was collected in grams and data points are representative of litter averages. Bar graph data are presented as means ± SE, significance was set at *p* < 0.05. Statistical analyses were determined using two-way ANOVA to examine the main effects of exercise and sex, followed by Tukey post hoc analyses. * denotes significance *p* < 0.05. Bold text denotes significant effect for that factor by two-way ANOVA.

Access to running wheels 10 weeks prior to conception and throughout pregnancy decreased male and female fetal weights. Figure 2 shows that placental weights of male and female fetuses at dpc 18.5 were not different due to maternal exercise, nor were there sex differences. However, there was an overall main effect of maternal exercise on fetal weights (*p* = 0.01) with reduced fetal weights in females (*p* = 0.05) and a trend towards reduced weight in males (*p* = 0.08) at dpc 18.5. There was a main effect of maternal exercise (*p* = 0.004) for decreased placental efficiency that was driven by the reductions in fetal weights and a significant reduction in males (*p* = 0.01) and a similar decrease in females (*p* = 0.06) via pairwise comparisons.

### 2.3. Placenta Microarray, GSEA, and IPA Results

**Figure 3:**
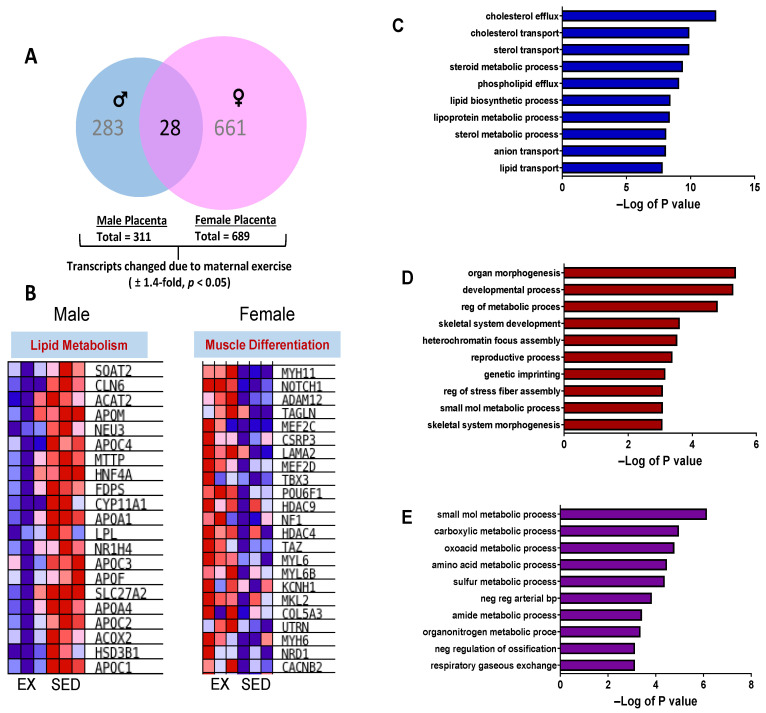
(**A**) Venn diagram indicating sex-specific and intersecting transcripts changed due to exposure to maternal exercise in placenta tissue. Gene expression was assessed in placenta at dpc 18.5 using GeneChip Mouse Genome 230 2.0 microarrays (N = 6 placentas per group). Genes were filtered based on a minimum ± 1.4-fold change (SED vs. EX) and *p*-values < 0.05 using Student’s *t*-test. (**B**) Gene Set Enrichment Analysis (GSEA) of transcripts related to lipid metabolism in male placentas and muscle differentiation in female placentas altered by maternal exercise. Red, white, and blue represent up-regulation, no relative effect, and down-regulation of transcripts, respectively. Enrichment of GO biological process terms up-regulated for (**C**) male placentas, (**D**) female placentas, and (**E**) intersection between male and female.

There were EX- and sex-specific changes in the expression of genes from placental tissue from male and female fetuses. Comparing the effect of sex (Female vs. Male) within each treatment group, 56 transcripts were differentially expressed in the EX group, and 42 differentially expressed genes (DEGs) were altered in the SED group (±1.2-fold, *p* < 0.05, Supplemental Tables S14 and S15). In contrast, comparing the effect of maternal exercise relative to sedentary counterparts, there was a combined 944 transcripts that were differentially expressed in placentas from males and female fetuses (±1.4-fold, *p* < 0.05), suggesting a larger impact of maternal exercise on global gene expression in placentas (Figure 3A). Moreover, female placentas had ~2.3-fold greater transcripts that were differentially expressed due to maternal exercise than placentas from males (661 vs. 283 transcripts, Supplemental Tables S2 and S6). There were 28 transcripts that changed in placentas from both males and females (Supplemental Table S10). Gene Set Enrichment Analysis (GSEA) was performed to identify biological processes that were differentially regulated by exposure to maternal exercise in male and female placentas as shown in Figure 3B. Male placentas had a down-regulation of transcripts associated with lipid metabolism due to maternal exercise and included genes such as apolipoproteins C1-C4, lipoprotein lipase (*Lpl*), acyl-CoA oxidase (*Acox*), and cytochrome P450 (*Cyp*)*11a1*. In female placenta transcripts involved in muscle differentiation that included *Notch1* and *Hdac 4* and *9*, several myosin heavy- and light-chain isoforms were up-regulated in EX as compared to SED (Figure 3B).

Gene ontology of placentas from males corroborated GSEA of lipid metabolism as transcripts were enriched in pathways such as cholesterol efflux, cholesterol transport, lipoprotein metabolic process, and lipid and sterol transport (Figure 3C). Similarly, gene ontology analysis in female placentas also supported GSEA. Figure 3D shows gene enrichment for the GO biological processes: organ morphogenesis, developmental process, skeletal system development, heterochromatin focus assembly, and skeletal system morphogenesis that were up-regulated in female placentas due to maternal EX. The intersection of the 28 transcripts that were similarly changed in both male and female placentas was enriched by numerous metabolic processes (carboxylic, oxoacid, amino acid, sulfur, amide, and organonitrogen) and respiratory gaseous exchange by gene ontology analysis as shown in Figure 3E.

**Figure 4:**
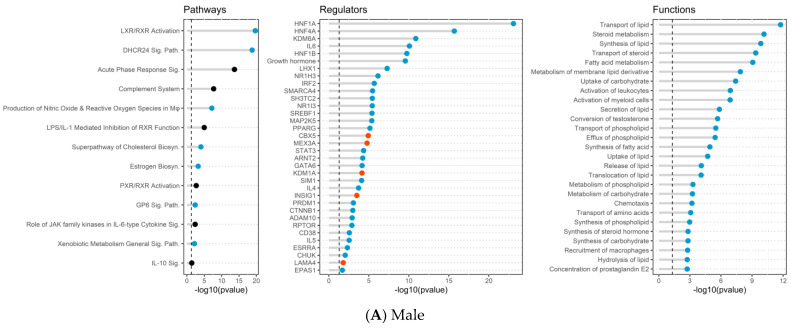

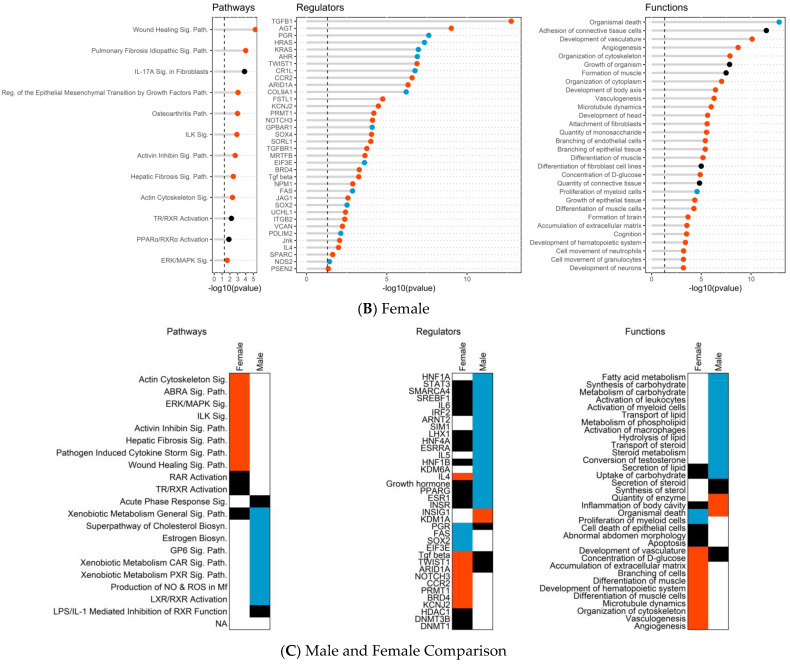
Top canonical pathways (CP), upstream regulators (UR), and biological functions (BF) of interest identified within (**A**) male DEGs and (**B**) female DEGs groups were identified in Ingenuity Pathway Analysis (IPA) and then graphed in R. Significance was set at a −log10(*p*-value) of 1.3 or greater as indicated by the vertical dashed lines and all entries were found to be significant. The red dots indicate activation of the CP, UR or BF entry with a z > 1.96. The blue dots indicated inhibition of the CP, UR or BF entry with a z < −1.96. The black dots indicate no significant z-score to determine directionality of the CP, UR or BF entry. (**C**) IPA results comparing exercise effects in females and males for biological functions (BF), signaling pathways (CP) and upstream regulators (UR). Heatmaps were created to examine the CPs, URs and BFs that were shared or different within IPA results between the two sexes. Red = Activation, z-score > 1.96, *p* < 0.05; Blue = Inhibition, z-score > −1.96, *p* < 0.05; Black = significant effect, *p* < 0.05, no z-score.

In addition, DEG lists for both male and female placentas were processed independently through Ingenuity Pathway Analysis (IPA), using canonical pathway (CP), disease and functions (DF), and upstream regulator (UR) analysis tools (Figure 4). Significant effects were revealed for each of the analyses (−log(*p*-value) of 1.3 or higher), indicating the over-representation of molecules in the DEG lists. If a given entry had sufficient information, then IPA reports directionally (activation/increase or inhibition/decrease), expressed as a z-score (|z| > 1.96 is considered significant). The reported results for UR analysis excluded exogenous chemicals, drugs, and toxins, focusing on biologically expressed molecules. The reported results from DF analysis excluded diseases, thereby focusing on “biological functions.” IPA also supported GSEA results, as male placentas from EX dams showed changes to URs and BFs related to lipid metabolism and steroid metabolism/transport (Figure 4A). Overall, IPA revealed 62 affected canonical pathways, 521 affected upstream regulators, and 472 affected biological functions in male placentas (−log10 (*p*-value) of 1.3 or greater). For example, the CP analysis, LXR/RXR activation, production of nitric oxide and reactive oxygen species in macrophages, superpathway of cholesterol biosynthesis, estrogen biosynthesis and xenobiotic metabolism displayed significant z-scores indicating inhibition (Supplemental Table S3, Figure 4A). For UR analysis, there was significant inhibition for HNF1A, HNF4A, KDM6A, IL6, NR1H3, NR1I3, SREBF1, PPARG, GATA6, and ESRRA (Supplemental Table S4, Figure 4A). The most prominently affected IPA functions included significant inhibition of a variety of functions related to lipid metabolism, steroid hormones and metabolism, fatty acid metabolism, and inflammation (Supplemental Table S5, Figure 4A).

In female placentas, IPA revealed 73 CPs, 639 URs, and 391 biological functions that were significantly affected in the females from EX compared to SED dam, including activation of wound healing, regulation of epithelial mesenchymal transition by growth factors, ILK signaling, activin inhibin signaling, hepatic fibrosis, actin cytoskeleton signaling, and ERK/MARPK signaling for CPs (Supplemental Table S7, Figure 4B). Some of the top URs included significant activation of TGFB1, AGT, TGFBR1, JAG1, and NTOCH3, and inhibition of PGR, KRAS, AHR, EIF3E, and FAS (Supplemental Table S8, Figure 4B). The most prominent biological functions identified related to angiogenesis, muscle growth and formation, endothelial cell branching, microtubule dynamics and brain function were significantly activated in female placentas from an EX dam compared to SED (Supplemental Table S9, Figure 4B). Combined with the female placenta exposed to maternal EX, we found activation of signaling pathways and functions related to muscle growth, brain, vascular development, and growth factors confirming the GSEA analysis results.

Finally, we utilized IPA comparison analyses to directly compare the altered pathways, regulators, and functions in males and females when comparing the effect of maternal EX (Figure 4C, Supplement Tables S11–S13). From these analyses, it was evident that differences due to maternal EX were mostly unique to each sex, while showing no effect in the other sex, or only significant effects but no directional information. For example, lipid metabolism and steroid metabolism were specifically affected in males, with no change in females. While females showed changes related to muscle growth, vascular development, and growth factors, these were not changed in males.

### 2.4. Placental mRNA Expression of Lipid Transport and Developmental-Related Genes

**Figure 5:**
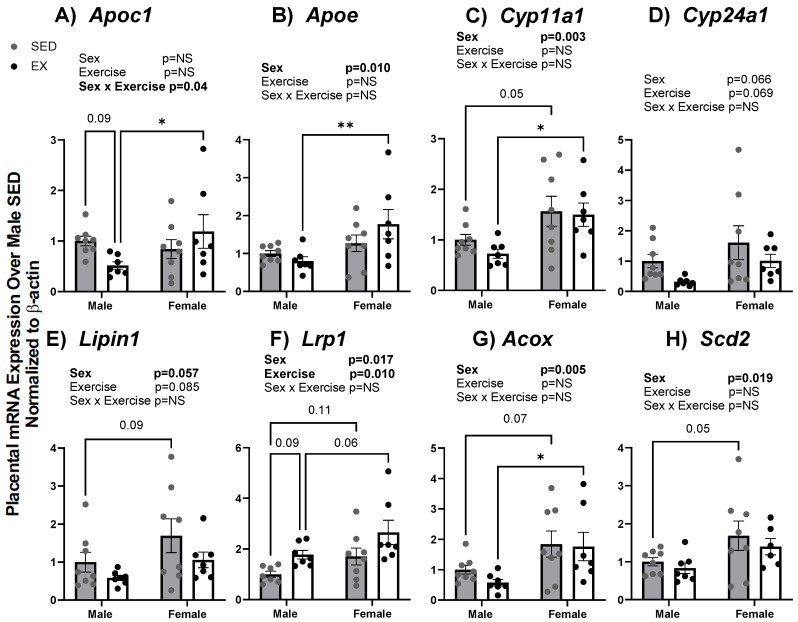
(**A**–**H**) mRNA expression of genes associated with lipid transport in placenta tissue of fetuses from SED and EX dams at dpc 18.5. Gene expression was assessed via quantitative real-time PCR. Bar graph data are presented as means ± SE, significance was set at *p* < 0.05. Statistical analyses were determined using two-way ANOVA to examine the main effects of exercise and sex, followed by Tukey post hoc analyses. * denotes significance *p* < 0.05, ** denotes significance *p* < 0.01. Bold text denotes significant effect for that factor by two-way ANOVA.

**Figure 6:**
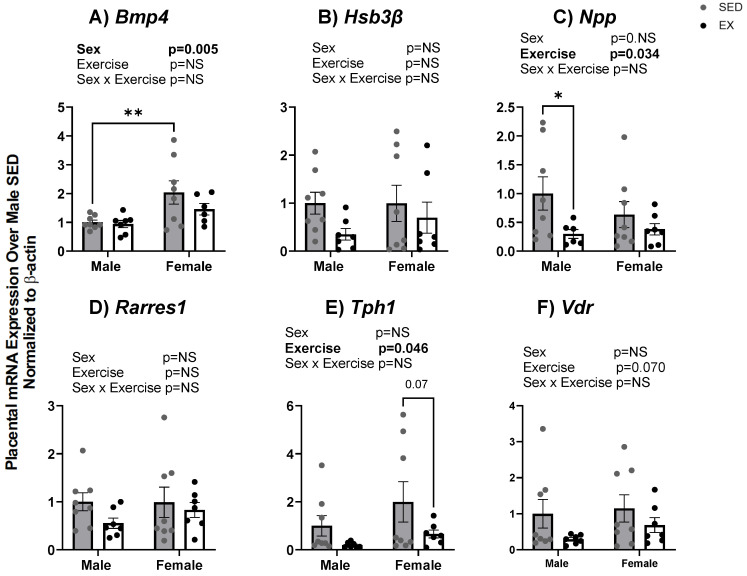
(**A**–**F**) mRNA expression of genes associated with development in placenta tissue of fetuses from SED and EX dams at dpc 18.5. Gene expression was assessed via quantitative real-time PCR. Bar graph data are presented as means ± SE, significance was set at *p* < 0.05. Statistical analyses were determined using two-way ANOVA to examine the main effects of exercise and sex, followed by Tukey post hoc analyses. * denotes significance *p* < 0.05, ** denotes significance *p* < 0.01. Bold text denotes significant effect for that factor by two-way ANOVA.

We confirmed results from microarray analysis using RT-qPCR for lipid transport and developmental-related transcripts. Similar to the microarray findings, mRNA expression of several lipid and cholesterol gene targets was decreased by maternal EX as compared to maternal SED and decreased only in male placentas. These targets included *ApoC1, Cyp11a1*, *Cyp24a1*, and *Acox* as shown in Figure 5A,C,D,G. mRNA expression of *Cyp11a1*, *Lrp1*, *Acox*, and *Scd2* were all increased in female placentas in EX and SED supporting the microarray transcript changes. In addition, we also revealed significant main effects for sex for *Apoe*, *Cyp11a1*, *Lipin1*, *Acox*, and *Scd2* transcripts. *Lrp1* showed both sex and exercise effects on mRNA expression while *Apoc1* showed a significant interaction effect (Figure 5). These findings suggest lipid transport genes appear to be affected more by sex than intrauterine exposure to maternal exercise. Developmental-related genes were also assessed in male and female placentas from EX and SED dams. Figure 6C,E demonstrate a significant exercise-induced effect with reductions in mRNA expression of *Npp* (*p* = 0.03) and *Tph1* (*p* = 0.04). Moreover, *Bmp4* mRNA expression showed a significant sex effect (*p* = 0.005) with increased expression levels in females compared to male placentas as shown in Figure 6A. There was also a slight reduction in *Hsd3β* due to maternal exercise only in male placentas (*p* = 0.11) (Figure 6B).

### 2.5. Fetal Liver mRNA Expression of Lipid Metabolism and Transport Genes

**Figure 7:**
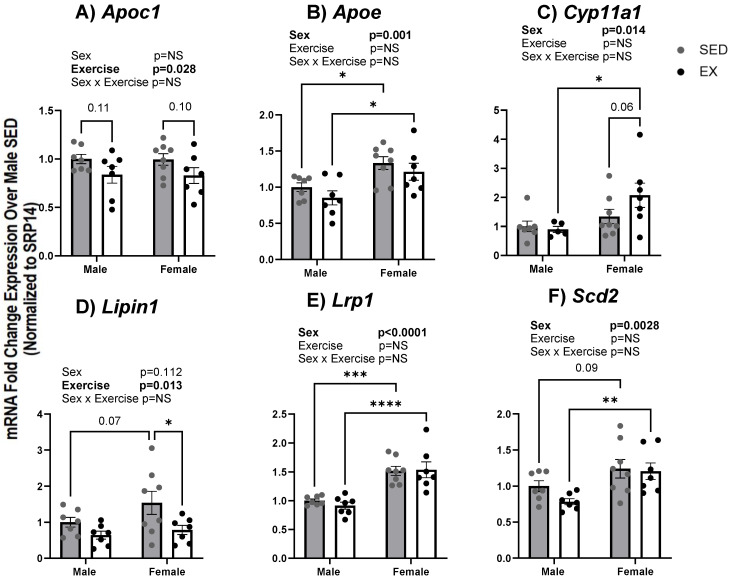
(**A**–**F**) mRNA expression of genes associated with lipid metabolism and transport in fetal liver tissue of fetuses from SED and EX dams at dpc 18.5. Gene expression was assessed via quantitative real-time PCR. Bar graph data are presented as means ± SE, significance was set at *p* < 0.05. Statistical analyses were determined using two-way ANOVA to examine the main effects of exercise and sex, followed by Tukey post hoc analyses. * denotes significance *p* < 0.05, ** denotes significance *p* < 0.01, *** denotes significance *p* < 0.001, **** denotes significance *p* < 0.0001. Bold text denotes significant effect for that factor by two-way ANOVA.

We also assessed mRNA expression in fetal livers from males and females from EX and SED dams. The effects on mRNA expression observed in the placenta were similar to fetal liver. However, some tissue-specific changes in mRNA expression were also apparent. Similar to the placenta lipid metabolism and transport genes, *Apoe* (*p* = 0.01 SED and *p* = 0.01 EX), *Lrp1* (*p* = 0.0002 SED and *p* < 0.0001 EX), and *Scd2* (*p* = 0.09 SED, *p* = 0.007 EX) were increased in the fetal liver of females from both EX and SED as compared to male fetal livers (Figure 7B,E,F). An exercise-induced decrease in fetal hepatic mRNA expression of *Apoc1* (Ex effect *p* = 0.028) and *Lipin1* (Ex effect *p* = 0.013) occurred in placentas from both males and females (Figure 7A,D).

## 3. Discussion

Exercise during pregnancy has been shown to be beneficial for both mother and child and may reduce the negative consequences of maternal obesity [15]. The placenta, which plays an essential role in nutrient transport and fetal growth, shows sexually dimorphic differences with maternal stressors, such as undernutrition and maternal obesity [38,39]. In this study, we investigated the influence of maternal EX on the placenta and potential mechanisms associated with maternal EX contributing to intrauterine effects, independent of maternal diet and/or weight changes in control-fed mice. A key finding from these studies was that maternal exercise induced sex-, and tissue-specific changes in the transcriptome of placentas and fetal livers at dpc 18.5.

One salient finding of this study was that the majority of transcripts altered by EX were influenced by the sex of the placenta. In fact, only 28 transcripts were changed in both male and female placentas due to intrauterine exposure to maternal EX. Maternal body weights were not different between EX and SED dams during the 10 weeks of wheel running prior to conception, allowing for the effects of maternal exercise to be examined independently of maternal weight changes. Previous studies have shown that maternal obesity and high-fat diets induce similar sexually dimorphic alterations in the placenta [40,41]. Additionally, when examining fetal livers at dpc 18.5, we identified lipid metabolism and transport genes similar to the placenta that were increased in females following EX compared to male livers and tissue-specific changes in mRNA expression levels. Collectively, these findings suggest that the effects of maternal EX on the placenta and presumably on the offspring are influenced by maternal habitus and are sexually dimorphic, highlighting the role of sex differences in developmental programming and metabolism.

There were several striking features of sexual dimorphic differences induced by maternal exercise on placental gene expression. Broadly, females had a much higher number of transcripts that were altered by maternal exercise than males, with 661 transcripts versus 283 transcripts, respectively. These transcripts were also differentially enriched in key biological pathways and functions. First, male placentas showed an inhibition of signaling pathways, biological functions, and specific sex hormone receptors (AR, ESR1, ESR2, ESRRA) and down-regulation of transcripts related to lipid and steroid metabolism following maternal EX. Second, there was an activation of signaling pathways and functions related to muscle growth, vascular development, and growth factors and up-regulation of transcriptions involved in muscle differentiation in female placentas only with maternal EX. Third, our studies suggest an inhibition of upstream X-linked regulator, KDM6A, in male placentas, and epigenetic regulators DNMTs (DNMT1 and DNMT3B) and HDAC1 as significant upstream regulators in female placentas and not male placentas.

A notable finding from these studies was the inhibition of lipid- and steroid-metabolism-related gene expression, pathways, and biological functions of the male placenta exposed to MAT EX and not the female placentas. More specifically, at the gene level, we found a significant reduction in the expression of *Hsd3β*, an enzyme that has an important role in steroid production, in male placentas with maternal EX. These results are in line with other clinical studies and animal models that have shown improved circulating lipids and lipid metabolism within adipose and liver tissue of offspring with maternal EX [24,42,43,44]. Previous studies have also shown sex differences in lipid metabolism between males and females. For example, enhanced lipid synthesis has been shown in females as compared to males in the context of in utero exposure to maternal obesity [45]. In addition, during exercise, females have increased utilization of muscle triglycerides which allows for greater utilization of lipids [46,47]. Pregnancy is also associated with remarkable changes in lipid metabolism and both lipids and lipoproteins are thought to be critical in the transport of lipids from mother to fetus and optimal for fetal growth [48,49,50]. Our findings are also in line with previous research showing sexual dimorphic effects in the placenta for both oleic acid and n-3 fatty acids [51,52]. Furthermore, steroid hormone receptors (ESR1 and PGR) identified to be inhibited in male placentas with maternal EX in the present study have also been shown to have an important role in placental development and energy metabolism [53,54]. Additionally, our results indicate inhibition of HNF4A in male placentas with maternal EX. HNF4A is a transcription factor that is known to play a role in sex differences in liver gene expression, has a critical role in fatty acid and cholesterol metabolism [55] and may also be influenced by sex hormones such as estrogen [56,57]. This further supports the inhibition of lipid metabolism processes as well as decreased expression of *Apoc1* and *Apoe* in the placenta of males compared to females with maternal EX. Together, these results provide evidence that lipid and steroid metabolism regulation with maternal exercise in the placenta differs with sex and is independent of dietary intake.

Sex steroids and changes in lipoproteins have also been previously associated with changes in placental efficiency and fetal growth [58,59]. These findings may partially explain the exercise-associated effect on fetal weights and placental efficiency identified in this study. Although the fetal/placenta ratio was decreased, it is important to note that maternal exercise did not induce changes in placental weight between males and females. The decreased ratio was completely driven by the reductions in fetal weight. However, this reduction in fetal weights and placenta efficiency has been previously shown by others with maternal exercise during pregnancy with both control and high-fat diets [60,61]. However, it is possible that placenta weights differed earlier in pregnancy, and by dpc 18.5, weights had normalized, as reported by others [62]. The reduced fetal weight is likely a reflection of the increased caloric demands associated with exercise; however, it is unclear whether this is a detriment or benefit to the developing fetuses.

Collectively, these changes in lipid metabolism and steroid metabolism of male placentas with maternal exercise could point to a potential mediating role that maternal exercise has on the placenta. The placenta is a key regulator of fetal growth and health, which occurs in large part through nutrient transport through the placenta. Among the key substrates that are transported through the placenta are lipids and fatty acids. In the context of maternal obesity, exercise during pregnancy can decrease excess lipids in the placenta [63]. In our results, we noted an inhibition of lipid metabolism and steroid metabolism in male placentas but not females. The significance of these changes remains to be studied in detail. However, the findings may suggest that some of the sexual dimorphic effects seen in the placenta may extend to changes in nutrient transport in the placenta, although additional studies would be needed to test this hypothesis.

In female placentas, maternal EX was associated with activation of signaling pathways and functions related to muscle growth, vascular development, and growth factors, and up-regulation of transcripts involved in muscle differentiation but not seen in male placentas. The transforming growth factor-beta family is known for its role in placentation and establishing the fetal–maternal interface [64,65]. In other tissues such as kidneys and muscles, aerobic exercise has been shown to decrease TGF-B expression [66,67] and increase VEGF both at the protein and mRNA levels compared to sedentary controls [68]. In addition, factors such as sex hormones and Y-chromosome-linked factors can also alter the expression of TBF-B family members [69]. Specifically, in this study, we identified the TGF-B family members activin, BMPs, IGF1, EGF, inhibin, TGFB and VEGF that were activated and/or significant upstream regulators specifically in female placentas. We also observed at the mRNA level altered expression of *Bmp4*, which is also associated with obesity and energy metabolism, to reveal a significant sex effect, where levels were increased in females compared to males. In relation to muscle growth and vascular development, both factors have been previously shown to be altered with exercise and have shown sex-specific differences, similar to our results [63,70,71,72]. These results not only show potential mechanisms on how maternal exercise independent of dietary intake is improving the health of the placenta and offspring, but those exercise effects are different depending on the sex of the placenta/offspring.

Another interesting observation from this study was the potential epigenetic regulators identified by IPA upstream regulator analysis. In male placentas from EX dams, we found inhibition of X-linked KDM6A as an upstream regulator in male placentas, but it was absent in females. In females, we identified significant upstream regulators; DMNT1, DNMT3B, and HDAC1 were regulated in female placentas with maternal EX but were not present in male placentas. KDM6A is a demethylase encoded by a gene with female-biased expression due to its escaping X-inactivation and function as an eraser of the repressive H3K27me3 mark [73]. In a clinical study of both males and females, mRNA expression of KDM6A in both skeletal muscle and myotubes was reduced in males compared to females, similar to our results [74]. We also know that sex-determining genes on the allosomes such as KDM6A can initially drive sex differences via genetic and epigenetic mechanisms. Another important epigenetic regulator is DNA methyltransferase enzymes (DNMTs), which are important for placental development and function and shown to differ in expression levels of blastocysts by sex [41,75,76,77,78]. It is hypothesized that sex differences in DNMT expression could be due to sex hormones, such as progesterone and estrogen [41,79,80] and altered by maternal nutrition status (obesity and/or nutrient restriction). Taken together, these differences in epigenetic regulators identified via pathway analysis suggest a potential epigenetic mechanism affected by maternal exercise and contributing to placenta sexual dimorphism. More detailed mechanistic studies are warranted to examine these pathways further.

It is important to note the limitations of the current study. First, the use of microarray analyses instead of RNA-sequencing was used on placenta tissue. We acknowledge that RNA-seq is a much more robust and sensitive method compared to microarray; nevertheless, this method was used to conduct these analyses at the time. However, the data still provide several novel findings to be followed up using RNA-seq and other methods. To improve the rigor of our studies, we employed three different analyses of DEGs and were able to find similar pathways and biological functions across all three platforms. A second limitation of this study is that we are not able to isolate the effects of preconception versus gestation exercise effects, as our maternal exercise intervention commenced prior to conception including both exercise during the pre-conception and gestation time. A third limitation is that no follow-up in offspring at later time points was conducted except for fetal livers at dpc 18.5. However, the primary objective of these experiments was to study the effects on the placenta, and therefore, offspring were not studied. Nonetheless, follow-up studies examining the long-term effects of maternal exercise on offspring health and development would provide beneficial information on exercise’s effects on developmental programming.

In conclusion, our results describe molecular changes in the placenta associated with maternal EX, which show pronounced sexual dimorphism. Our study identified novel pathways, functions and upstream regulators that are differentially regulated in the placenta of males compared to females. These results provide potential mechanisms for future studies to elucidate sex-specific developmental programming associated with prenatal exercise.

## 4. Materials and Methods

### 4.1. Animals and Chemicals

Virgin female C57BL/6 mice were obtained from the Jackson Laboratory (Bar Harbor, ME, USA). Animals were housed in an AAALAC-approved animal facility at the Arkansas Children’s Research Institute in a temperature- and light-controlled room (12 h light/12 h dark cycle). All experimental protocols were approved by the Institutional Animal Care and Use Committee at the University of Arkansas for Medical Sciences. Unless specified, all chemicals were obtained from Sigma-Aldrich Chemical Co., (St. Louis, MO, USA).

### 4.2. Experimental Protocol

Six-week-old female C57BL/6 mice were divided into 2 groups: with (exercise, EX) or without (sedentary, SED) access to voluntary running wheels. EX was provided via 24 h access to wheels for 10 weeks prior to conception until late pregnancy (18.5 days post coitum). Each female mouse was individually housed and allowed ad libitum access to a purified AIN-93G (10% kcals from fat) diet [81,82]. Running distances were recorded daily using a Sigma Sport 16.12 bicycle odometer (Sigma Sport Ltd., Neustadt an der Weinstraße, Germany) and food intake was measured 3 times per week. Magnetic Resonance Imaging (MRI) was performed using EchoMRI (EchoMRI LLC, Houston, TX, USA) on non-sedated dams at 6 and 10 weeks of wheel running, prior to conception. Each female was mated with a lean male breeder; presence of a vaginal plug was recorded and designated as 0.5 days post coitum (dpc). At dpc 18.5, N = 7 SED and N = 8 EX dams were sacrificed and individual placentas and fetuses from each dam were collected and weighed and immediately frozen for later analyses. Sex was determined for all placentas via qPCR for the Sry gene (Supplemental Table S1). Livers were excised, weighed, and immediately frozen for later analyses. Non-fasted blood was also collected via cardiac puncture from all dams at the time of tissue collection. Blood was centrifuged (10,000× *g* @ 4 °C for 10 min) to obtain serum and was stored at −20 °C. Serum insulin was measured using an ELISA kit following the manufacturer’s protocol (Millipore, St. Louis, MO, USA). Triglycerides (Cayman Chemical, Ann Arbor, MI, USA), Total Cholesterol (Fujifilm Healthcare, Lexington, MA, USA), Glucose (Fujifilm Healthcare, Lexington, MA, USA), and non-esterified fatty acids (NEFA, Fujifilm Healthcare, Lexington, MA, USA) were assessed in the serum by colorimetric assays following standard procedures.

### 4.3. Microarray Analysis

Total RNA from individual whole placentas was isolated using TRI Reagent and purified using RNeasy columns (Qiagen, Valencia, CA, USA) including on-column deoxyribonuclease digestion. Six microarrays (GeneChip Mouse Genome 230 2.0, Affymetrix, Santa Clara, CA, USA) were used for each group, representing biologically distinct pools of RNA from placentas from different dams. Briefly, 5 μg of purified RNA was used to synthesize first- and second-strand cDNA. Labeled aRNA (amplified RNA) was synthesized from double-stranded cDNA using the GeneChip IVT labeling kit (Affymetrix, Santa Clara, CA, USA) according to the manufacturer’s protocol. The probe array was scanned with a GeneChip Scanner 3000 (Affymetrix) and .CEL files were generated using the GeneChip Command Console (Affymetrix). Data analysis was performed using functions in the affy, limma and sva packages in R [83,84]. CEL files were processed using RMA algorithm for background adjustment, normalization, and log2-transformation of perfect-match values [83,84]. Genes were filtered based on ±1.4-fold change and *p*-value < 0.05 (Student’s *t*-test) between EX and SED dam offspring. Enrichment of gene ontology (GO) terms for differentially regulated genes was performed using GoRilla [85]. Gene Set Enrichment Analysis (GSEA) was utilized to independently identify biological processes enriched by maternal exercise. GSEA does not rely on an arbitrary cutoff (such as fold change between groups) and is a computational method that determines whether a set of genes defined a priori shows statistical significance or concordant differences between two biological states.

In addition, we performed Ingenuity Pathway Analysis (IPA) analyses between the male and female placenta differentially expressed gene (DEG) lists (QIAGEN Inc., Germantown, MD, USA, https://www.qiagenbioinformatics.com/products, accessed on 18 July 2023). QIAGEN (IPA; Hilden, Germany) is a unique program that provides a breadth of analysis options compared with other programs [86]. We chose to analyze our data through this database as well as GSEA because IPA derives results via a knowledge base that contains more than 7 million research findings. IPA result categories are also much more specific, and the directions of changes for pathways and functions can be determined. Lastly, IPA can reveal affected upstream regulators, an output that is relevant to identifying and pursuing testable hypotheses to determine underlying mechanisms.

### 4.4. Real-Time Quantitative RT-PCR (qPCR)

Total RNA was isolated from mouse placenta or fetal liver at dpc 18.5 (N = 7 SED, N = 8 EX) using RNeasy mini columns (QIAGEN, Valencia, CA, USA) including on-column DNase digestion. One microgram of total RNA was reverse transcribed using iScript cDNA synthesis kit (BioRad, Hercules, CA, USA). Real-time PCR analysis was performed as described previously using an Applied Biosystems Prism 7500 Fast instrument (Carlsbad, CA, USA). Gene-specific primers were designed using Primer Express Software v3.0 (ThermoFischer, Waltham, MA, USA, Supplemental Table S1). Relative amounts of mRNA were quantified using a standard curve and normalized to the expression of β-actin for placental gene expression and *Srp14* for liver gene expression (Supplemental Table S1).

### 4.5. Statistical Analysis

For all placenta and fetal weight calculations, we took the average of all placentas and/or fetus from each individual dam. Then, the average for each dam was used for statistical analyses. Data are expressed as means ± SEM, significance was set at *p* < 0.05. Data were analyzed by two-way ANOVA to determine the significant effects of exercise, sex, and the interaction of sex and exercise followed by post hoc analysis using Tukey’s multiple comparison tests, *p* < 0.05 was considered statistically significant. Statistical analyses were performed using GraphPad v.10.0 software (GraphPad Software Inc., Boston, MA, USA).

## Figures and Tables

**Table 1:** Dam characteristics and serum parameters at dpc 18.5.

	SED	EX
Body wt (g)	24.2 ± 1.1	24.9 ± 0.4
Liver wt (g)	1.7 ± 0.09	1.9 ± 0.04
% Liver wt	7.3 ± 0.04	7.7 ± 0.01
Fat pad wt (g)	0.7 ± 0.05	0.9 ± 0.12
% Fat wt	2.8 ± 0.02	3.0 ± 0.01
Total Litters	8	7
Total Pups/Litter	7.1 ± 0.3	8.3 ± 0.5
Male:Female Ratio	2.6 ± 0.7	1.8 ± 0.7
Insulin (ng/mL)	1.5 ± 0.2	1.7 ± 0.2
Glucose (mmol/L)	13.8 ± 0.8	13.7 ± 0.4
Triglycerides (mg/dL)	50.7 ± 2.2	* 61.1 ± 3.3
NEFA (meq/L)	0.39 ± 0.09	0.42 ± 0.09
Cholesterol (mg/dL)	56.2 ± 4.5	70.2 ± 5.4

Body, liver, and fat pad (retroperitoneal plus gonadal fat depots) weights were assessed at the time of sacrifice. Percent liver and fat weight were calculated as tissue weight in grams divided by total body weight in grams. Data are expressed as mean ± SEM. Differences between dams were analyzed using Student’s *t*-test, significance was set at *p* < 0.05, * denotes significance.

## Data Availability

Placental microarray data are available at the Gene Expression Omnibus (GSE243228). IPA data sets and DEG lists are in Supplemental Tables.

## Supplementary Materials

The following supporting information can be downloaded at: https://www.mdpi.com/article/10.3390/ijms242216441/s1.
